# Canada’s Student Mental Health Network: Protocol for a Comprehensive Program Evaluation

**DOI:** 10.2196/41521

**Published:** 2023-06-22

**Authors:** Amy Ecclestone, Brooke Linden, Caitlin Monaghan, Sally Zheng

**Affiliations:** 1 Health Services and Policy Research Institute Queen's University Kingston, ON Canada; 2 Department of Public Health Sciences Queen's University Kingston, ON Canada; 3 Temerty Faculty of Medicine University of Toronto Toronto, ON Canada; 4 Department of Psychology Toronto Metropolitan University Toronto, ON Canada

**Keywords:** health promotion, higher education, mental health, postsecondary, program evaluation, stress, students, university, well-being

## Abstract

**Background:**

Prevalence estimates for mental health–related problems, including above-average stress, psychological distress, and symptoms of mental illnesses have increased significantly among Canadian postsecondary students. As demand for downstream mental treatment has surpassed many institutions’ abilities to deliver timely care, there is a need for innovative upstream supports that foster mental health promotion and mental illness prevention among this population.

**Objective:**

Supported by an extensive network of student volunteers, Canada's Student Mental Health Network is a virtual, one-stop shop for centralized mental health education and evidence-based resources tailored to postsecondary students. This article describes a protocol for the comprehensive evaluation of the Network.

**Methods:**

Development of the Network was developed using a participatory action research framework. Network content is created and curated by students and reviewed by subject matter experts. The proposed program evaluation will include both a formative process evaluation and a summative impact assessment to determine the feasibility, acceptability, and utility of the Network in addition to assessing change in the 3 primary outcomes of interest: mental health literacy, perceived social support, and help-seeking behavior. Participants will be recruited directly from the Network website using a “rolling” recruitment approach to allow for continuous data collection and evaluation. A combination of qualitative (ie, interviews) and quantitative (ie, surveys) methods of data collection will be used.

**Results:**

The process of evaluation of the Network will begin in September 2022, collecting data for 1 year. In September 2023, the impact evaluation will begin using the same follow-up schedule. Data collection will then remain ongoing to facilitate the continued evaluation of the Network. Reports detailing evaluation data will be released annually.

**Conclusions:**

The Network is a novel and innovative method of delivering universal mental health promotion to Canadian postsecondary students by providing centralized and freely accessible mental health education and resources, created by students and validated by subject matter experts. The continued creation and curation of resources for the Network will be ongoing to meet the evolving needs of the target population.

**International Registered Report Identifier (IRRID):**

PRR1-10.2196/41521

## Introduction

### Background

Prevalence estimates for above-average stress, psychological distress, and symptoms of mental illnesses have increased significantly among Canadian postsecondary students over the past several years [1]. Excessive stress has been linked to a number of negative academic (ie, decreased performance and decreased motivation) and health (ie, anxiety and depression) outcomes among students [2,3]. Indeed, data collected from Canadian postsecondary institutions through the 2019 National College Health Assessment II survey revealed large proportions of students feeling hopeless (63.6%), overwhelmed (88.2%), and anxious (68.9%) within the past 12 months [4].

Existing research has highlighted the variety of stressors students experience within the postsecondary setting [5,6]. The COVID-19 pandemic produced additional, novel stressors, with national data suggesting postsecondary aged Canadians were the most likely to report increased symptoms of anxiety and depression throughout the pandemic [7]. Over the past decade, demand for downstream mental health services (ie, counseling and pharmacotherapy) has surpassed institutions’ capacities to deliver timely care [8]. In the wake of the pandemic and its mental health-related fallout, there is reason to believe that this demand will only continue to increase [9]. Placing additional focus on upstream mental health supports (ie, those that aim to intervene before the development of psychological distress) may contribute toward alleviating the bottleneck currently observed at the downstream level. Upstream supports can help to bolster students’ resilience and well-being by providing them with the skills and resources required to monitor and respond to the changes in their mental health, as well as providing education around appropriately aligning level of help seeking with level of need.

While some form of mental health promotion is offered by many institutions, a national review of mental health and well-being services on Canadian postsecondary campuses found that only 70% of respondents felt students were well-informed about available resources on campus, and about mental health issues in general [10]. Many respondents (84%) also indicated that they felt there was room for improvement in the mental health promotion supports currently offered on their campus [10]. One of these barriers is the cost associated with accessing some forms of mental health education. For example, many institutions now offer for-credit mental health courses, but students must be formally registered at a substantial cost. Training courses such as Mental Health First Aid, SafeTALK, and others sometimes also require a registration fee. The second barrier lies in the reality that some institutions have more available budget than others to put toward campus mental health supports. Larger schools tend to have more funding and can therefore offer more robust mental health support to students compared to smaller schools. At most institutions, the majority of the mental health budget is skewed toward downstream services, typically leaving upstream supports to operate on more limited resources. Finally, accessibility and logistics barriers are another consideration. Many mental health promotion supports provided by institutions take the form of on-campus workshops and events. This poses a barrier for some groups of students who may be left out (ie, web-based students, students studying abroad, and students who commute to campus), as well as students with multiple competing priorities to manage (ie, coursework, extracurricular activities, sports, and employed work). These observed gaps demonstrate a need for cost free, universally accessible mental health promotion.

### Introducing Canada’s Student Mental Health Network

Canada’s Student Mental Health Network (hereafter, “the Network”) is a virtual, one-stop shop for mental health education and evidence-based resources, tailored specifically to postsecondary students. Development of the Network’s website began in 2020, using a participatory research design wherein members of the target population work alongside researchers as contributors, helping to provide a deeper understanding of their thoughts, needs, and experiences [11]. In line with this approach, all content on the Network is created and curated by students and reviewed for validity by subject matter experts (ie, researchers and health care practitioners) using a collaborative approach to the cocreation and translation of knowledge. Based on the extant literature consistently highlighting mental health education, social support, and help seeking as major predictors of positive mental health outcomes for students, the Network targets these 3 areas as primary objectives: (1) to improve mental health literacy; (2) to encourage the development of strong social support networks; and (3) to improve awareness of available resources and encourage appropriate help seeking. The content on the website is aligned with these objectives, centered around 3 pillars: learn, connect, and access.

#### Learn

Mental health literacy (MHL) refers to “understanding how to obtain and maintain positive mental health; understanding mental illnesses and their treatments; decreasing stigma related to mental illnesses; and enhancing help-seeking efficacy (knowing when and where to seek help and developing competencies designed to improve one’s mental health care and self-management capabilities)” [12]. MHL has been identified as a foundational component in mental health promotion and mental illness prevention [12]. Improved mental health education not only enables an individual to recognize changes in their mental health and well-being, but also empowers them to respond appropriately [13,14]. Given that MHL is context specific, it is important for resources to be tailored appropriately to the target population (ie, students) and the postsecondary setting [14]. The *learn* section of the Network facilitates access to free and evidence-based mental health education. Education is provided in an interactive, accessible, and aesthetically appealing format. Some features in this section include: a series of web-based mental health education modules, created by Network volunteers and validated by subject matter experts; a curated library of informational and educational TedTalks; a library of free, web-based mental health courses; and a library of detailed toolkits, providing guidance on topics including financial literacy, relationship management, and an introduction to cognitive-behavioral therapy techniques.

#### Connect

The development of strong social support networks is a key predictor of positive mental health outcomes and overall well-being across the course of life, but particularly during adolescence and early adulthood [15,16]. The majority of Canada’s postsecondary students are “emerging adults,” defined as ages between 18 and 25 years [16]. Students navigate a number of major transitions during this time, including the transition to a postsecondary setting, integration into a new social environment, and increased autonomy and responsibility. A lack of social support when faced with these adjustments can lead to social isolation, loneliness, and homesickness [17]. In fact, many students struggle to form new, meaningful friendships at university or college and can find it difficult to maintain pre-existing relationships, such as those with their parents or childhood friends [18,19]. Together, the breakdown of previous social ties and difficulty finding a new social support network at school can result in mental health deterioration [20]. Social support has also been found to be a protective factor against student burnout [21]. Burnout is common in students in academic settings and is linked to psychological distress and perceived stress [22,23].

The *connect* section of the Network encourages students to develop healthy social support networks by providing access to resources designed to encourage peer connection. Some features in this section include: a clubs portal, which provides single-click access to student club listings for every university and college across Canada; the Student Mental Health Network podcast, featuring interviews with subject matter experts and students on a variety of topics relevant to student mental health, including sharing lived experiences; and a library of web-based forums designed to foster peer connection.

#### Access

Commonly perceived barriers to help seeking for mental health-related problems among students include a preference for self-management [24], a lack of time [24,25], perceived stigma [26-28], and a lack of awareness around availability of resources [27]. Structural barriers also exist; students are sometimes surprised to learn of the mental health–related services and supports available to them through their institution and frequently report not knowing where to physically access them [29]. This is particularly evident on sprawling campuses, where health supports are split across several different buildings rather than in a centralized location [29]. Additionally, some students are predisposed to structural barriers as a result of socioeconomic status, race or ethnicity, or gender [30]. Increasing awareness of available services and supports both on and off campus, as well as educating students about aligning their level of mental health need with the level of mental health support sought, is essential to supporting the mental health and well-being of the postsecondary population [30].

Rather than provide services, the goal of the *access* pillar of the Network is to improve students’ awareness of existing resources and services available to them, as well as to improve education regarding appropriate levels of help seeking. These resources include: (1) a mental health resource bank, which includes one-click access to Student Wellness Services websites for every university and college across Canada, as well as a library of community mental health resources (both in person and web-based) at the provincial and national levels; (2) a library of evidence-based and physician recommended mental health mobile apps; (3) an interactive mental health services map detailing locations of mental health support on Canadian campuses across the country; and (4) a bank of stress management resources tailored specifically to postsecondary students based on the stressors measured by the Postsecondary Student Stressors Index [31].

## Methods

### Project Overview

The overall goal of the Network is to reduce barriers and facilitate universal access to upstream mental health supports for postsecondary students across Canada. This study describes a protocol for a comprehensive evaluation of the Network, including a formative process evaluation and a summative impact assessment. The key objectives of this evaluation are to determine the feasibility, acceptability, and utility of the Network in addition to assessing change in the 3 primary outcomes of interest aligning with website content: mental health education, perceived social support, and help-seeking behavior.

### Study Design 

The proposed program evaluation will include a formative process evaluation and a summative impact assessment. The logic model we have developed for the purposes of evaluating the Network can be found in Multimedia Appendix 1. Process evaluations are intended to assess 2 key components of program function: (1) whether the program is reaching the intended targeted population (*acceptability*) and (2) whether its delivery and function is consistent with program design specifications (*feasibility*) [32]. Impact assessments are intended to assess the observed change in the outcomes of interest and estimate the degree to which that change may have occurred in the absence of the intervention (*utility*) [33]. Alignment of evaluation design with initiative life span is imperative. For example, conducting an impact assessment when a program is too “young” for observable change to have occurred in outcomes of interest may lead to the incorrect conclusion that the program or initiative is not effective. Given that the Network is a newly developed initiative, we will begin with a formative process evaluation that focuses on the user experience in terms of feasibility and acceptability. A summative impact assessment will be conducted after the Network has been in operation for a minimum of 1 year.

Increasingly, mixed methods approaches to studying complex public health problems have been recommended, as the use of qualitative and quantitative methodologies in tandem provides a more holistic and thorough understanding than either approach individually [32]. A concurrent mixed methods study design will be used to facilitate a comprehensive evaluation of the Network, where both quantitative and qualitative data will be used to facilitate both depth and breadth of understanding.

### Measures

#### Process Evaluation

Data for the process evaluation phase of this project will be drawn from 2 sources: (1) Google Analytics and (2) cognitive interviews (Table 1). To collect information regarding the reach of the Network in terms of website engagement and user experience, we will analyze website metrics collected through Google Analytics. These metrics include total number of users, average number of users (weekly, monthly, and annually), average engagement time (time spent on each component of the website), user “stickiness” (average length of visits), page views, event counts (clicks and file downloads), and user region.

We will use individual cognitive interviews employing a “think aloud” technique [34] to further evaluate the user experience and highlight gaps in content. Participants will be asked to screen share over Zoom as they navigate through the Network website and talk us through their navigation, identifying content-related issues or preferences as well as any navigational challenges. The interviews will include questions related to sociodemographic characteristics, important website attributes, and perceived acceptability and feasibility (Table 1). Collecting this additional in-depth qualitative data on website attributes and navigation will augment the quantitative data collected through Google Analytics and allow us to identify gaps and areas for improvement to ensure the best possible user experience.

**Table 1:** Process evaluation measures.

Mode of data collection and component			Measures/item	Qualitative/quantitative
**Cognitive interviews**				
	Sociodemographic characteristics	Age Gender Ethnicity Year of study Level of study Area of study International student (Y/N)^a^ First generation student (Y/N) Identify as a visible minority (Y/N) Identify as a person with a disability (Y/N) Self-reported SES^b^ Grade Point Average Current self-rated mental health Mental health diagnoses (lifetime) Impact of COVID-19 on mental health Previous access of mental health supports and type		Quantitative/qualitative
	Website attributes	Appearance Content Interactivity Inclusivity and representation Accessibility		Qualitative
**Google Analytics**				
	Website engagement and user experience	Total number of users Average number of users (weekly, monthly, and annually) Average engagement time Page views User stickiness (average length of visits) Event counts (visits, clicks, scrolls, and file downloads) User location (country, region, and city)		Quantitative

^a^Y/N: yes/no.

^b^SES: socioeconomic status.

#### Impact Assessment Evaluation

Data for the impact assessment evaluation of the Network will be drawn from 2 sources: (1) web-based surveys and (2) interviews (individual and group) (Table 2).

The impact assessment survey will include questions related to sociodemographic information (eg, age, gender, ethnicity, year of study, level of study, area of study, and estimated grade point average) and important website attributes (eg, appearance, content, interactivity, inclusivity or representation, and accessibility). However, the survey will also include several validated instruments designed to evaluate change in each of the outcomes of interest (eg, scales evaluating mental health literacy, perceived social support, and help-seeking behaviors). The majority of the survey will consist of closed-ended, quantitative questions, with a few open-ended, qualitative response options. More in-depth descriptions of each of the selected measures and their associated psychometric properties can be found in Multimedia Appendix 2.

Web-based interviews will be held in both individual and focus group formats, depending on recruitment. A semistructured interview guide will be used to assess students’ perspectives on changes in the outcomes of interest as well individual understandings of mechanisms that may have contributed to the change (eg, improved mental health literacy due to completion of Network modules). Interviews will be used to evaluate students’ experiences engaging with the website, as well as their perceptions of potential mechanisms through which they observed change in their mental health education, connections with members of their social support network, and help-seeking attitudes and behaviors.

**Table 2:** Impact assessment evaluation measures.

Mode of data collection and component		Measure/item	Qualitative/quantitative
**Web-based survey**			
	Sociodemographic characteristics	Same as process evaluation	Quantitative/qualitative
	Mental health	Perceived stress scale, 4-item [35] Kessler Psychological Distress Scale, 6-item [36] Brief-postsecondary student stressors index [6]	Quantitative/qualitative
	Mental health literacy	Mental health literacy scale, adapted [37] Social distance subscale of OMS-WA^a^ [38]	Quantitative/qualitative
	Social support and resilience	Multidimensional scale of perceived social support [39] Connor-Davidson Resilience Scale (10-item) [40]	Quantitative/qualitative
	Help seeking	Barriers to care evaluation, revised [41] Self-stigma of seeking help scale [42] General help seeking questionnaire [43]	Quantitative/qualitative
**Interviews**			
	User experience and impact of website engagement	User experience Website navigation Perceived impact	Qualitative

^a^OMS-WA: Opening Minds Scale–Workplace Attitudes.

### Procedure

#### Participants and Recruitment Strategy

Canadian postsecondary students who have engaged with the Network are the target population for the proposed evaluation. Students attending any type of postsecondary institution (ie, college, university, etc) will be eligible to participate. Participants will be recruited through 3 possible avenues: (1) directly from the Network website through a posted call for participants; (2) through the Network’s social media channels; or (3) through a snowball sampling method. Network contributors will communicate the opportunity to become involved in the evaluation study through word-of-mouth as the first point of contact. To avoid bias, contributors themselves will not be eligible to complete the impact assessment survey. Participants who complete the survey will have the opportunity to refer 5 other students to complete it in exchange for 1 entry into our raffle draw per completed survey submitted by a referral.

**Figure 1:**
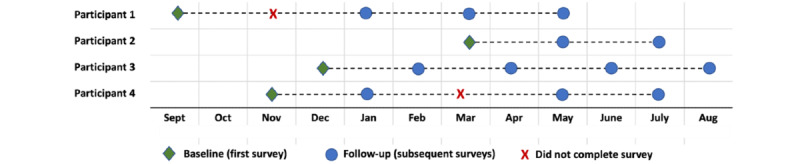
Visual depiction of rolling recruitment strategy.

The process evaluation, recruitment, and data collection period will take place between May 2022 and May 2023, allowing for a formative assessment of the first year of operation. A “rolling” recruitment approach (Figure 1) will be used for the impact assessment, allowing participants to complete the survey at any time within the data collection period of September 2023 to September 2024. This approach will allow for continued data collection as new users engage with the Network over time. Rolling recruitment approaches have been shown to reduce recruitment delays and improve statistical power by minimizing the effects of loss to follow-up [44]. Students may participate in process evaluation, impact evaluation, or both.

The appropriate sample size for the process evaluation interviews will be determined by the length of time it takes to reach saturation. For the impact assessment survey, we will aim to obtain a total sample size of 10,000 respondents. Statistics Canada data for the latest academic year (2020-2021) indicates that a total of 2.2 million students are enrolled in a postsecondary program [44]. Our goal will be to obtain responses from students across Canada, with relative contributions by region (Western ~30%, Atlantic ~5%, and Central Canada ~65%) in order to obtain a sample that is generalizable to the broader Canadian postsecondary population. We determined the relative contributions by region using Statistics Canada’s Postsecondary Student Education Dashboard [35].

#### Data Collection and Processing

Google Analytics data will be continually collected from the website over the lifetime of the Network for program monitoring purposes. Cognitive interviews will be conducted, recorded, and transcribed through Zoom [36]. Participants will be compensated for their participation in a cognitive interview with a US $14.97 electronic gift card to Amazon.

Web-based surveys will be administered through Qualtrics survey software [37]. A letter of information (LOI) for the evaluation will appear as the first page of the survey, communicating the purpose of the evaluation, survey content, incentives to participate, process for withdrawing submitted responses, data deidentification and confidentiality, and consent for use of data in future analyses. After reviewing the LOI, participants will indicate their informed consent before being granted access to the survey questions. The only identifying information on the survey will be students’ institutional email addresses used to link their responses across surveys. Emails will be permanently deleted and replaced with numeric participant numbers at the end of the data collection period (September 2024), effectively deidentifying data. The impact assessment survey will consist of approximately 30 close-ended questions and is expected to take around 30 minutes to complete. Following the submission of their first survey, participants will be invited to submit a brief follow-up every 2 months, enabling us to track their responses over time. Participants who “miss” a follow-up will continue to be invited to complete a survey every 2 months throughout the data collection period, unless they choose to opt out of receiving additional surveys. Data will be linked between survey iterations wherever possible.

Participants will be compensated for their submission of each survey with an entry into a prize draw for a chance to win 1 of several electronic gift cards. They will also be provided with an additional entry into the raffle for every individual they refer who completes the survey, per the snowball sampling strategy outlined above. Prize draws will be completed at the end of each academic term.

At the end of the survey, participants will be asked whether they would be interested in participating in an interview to further discuss their engagement with the Network. Participants who express interest will be contacted by email to indicate their preference for either a one-on-one interview or a focus group interview, depending on recruitment numbers. We will attempt to place students from the same region in focus groups together to encourage participants to draw on common experiences and promote idea sharing. Participants will be provided with a letter of information and asked to verbally consent to keeping discussions confidential as well as to interviews being recorded and transcribed. Individual interviews will be 1-hour in length, while the length of focus group interviews will depend on the number of participants included. All interviews will be facilitated over Zoom to accommodate collaboration with participants across Canada. Participants will be compensated for their participation in interviews with a US $20 electronic gift card to either Amazon or Starbucks.

#### Risk Mitigation

The risk associated with the proposed evaluation is low. However, to mitigate any feelings of elevated stress or emotional distress participants may feel after completing a survey, an interview, or both, we will refer participants to the access section of the Network which includes contact information for campus Student Wellness Services across the country as well as other evidence-based resources.

### Analysis

#### Process Evaluation

Quantitative data and website metrics collected through Google Analytics will be analyzed through the use of descriptive statistics (eg, frequencies, measures of central tendency, and dispersion) using R statistical software [38]. Cognitive interview data will be analyzed through the use of the problem codes displayed in Table 3, which were adapted from those proposed for use in the evaluation of new survey tools [39,40]. Following each interview, corrective action will be taken to assess the identified problems, ensuring that each interview provides maximum value to the improvement of the Network. In the event that a new issue arises that is not captured by existing problem codes, a new code will be added to the list for use in subsequent interviews. After problem codes have been applied, interview transcripts will also be coded using a thematic analysis framework [41,42]. A total of 2 research assistants will independently review interview data and generate initial codes, subsequently meeting to share interpretations and develop a codebook. A third researcher will be available to break ties where disagreements arise. Research assistants will then return to the data to generate initial themes and categories.

**Table 3:** Cognitive interviewing problem codes.

Problem code	Description	Corrective action
1. Clarity	Participant was confused by website content	Revise content
2. Relevance	Respondent felt that website content was not relevant to postsecondary student mental health and well-being	Revise content/remove content
3. Redundancy	Respondent felt that website content was repetitive or redundant, or could be combined with another content piece	Revise content/remove content
4. Bias	Respondent felt the presentation of website content was biased or leading in some way	Revise content/remove content
5. Missing	Respondent felt there was content missing from the website	Add content
6. Navigation	Respondent felt website content was difficult to navigate or locate	Optimize navigation/revise content organization
7. Accessibility	Respondent felt website content was not accessible	Optimize accessibility
8. Representation	Respondent felt website content did not represent all equity-deserving group	Optimize representation/revise content

#### Impact Assessment

Both descriptive (eg, frequencies, measures of central tendency, and dispersion) and inferential statistics will be calculated for quantitative data collected through the impact assessment survey using R statistical software. We will explore (1) relationships between sociodemographic variables and outcomes of interest (ie, mental health literacy, perceived social support, and help seeking), (2) the degree of change in outcomes of interest over time, and (3) the relationship between degree of engagement with the Network and degree of change observed in outcomes of interest. Trend analyses examining changes in stress, mental health, and the primary outcomes of interest over time will be conducted once enough data has been collected to facilitate these analyses.

Transcripts derived from individual and focus group interviews will be coded in NVivo [43] using a thematic analysis framework [42] using the approach described above. Qualitative interview data will be triangulated with participants’ quantitative survey data, with qualitative data used to provide more context and facilitate a more in-depth understanding of the user experience and the mechanisms of change in outcomes of interest.

### Ethical Considerations

The Tri-Council Policy Statement TCPS2 regarding ethical conduct for research involving humans states that Research Ethics Board (REB) approval is exempted in cases of program evaluation activities normally administered in the ordinary course of operation (Article 2.5) [45]. Moving forward, should the research team choose to conduct additional secondary data analyses to answer emergent research questions, REB approval will be sought. Despite the program evaluation being exempt from REB approval, participants will still be provided with a LOI to obtain their informed consent to participate through survey completion.

## Results

Phase 1 of this project entailed the development of the Network website, including the curation and creation of relevant resources for the web-based space. Moving forward, content developed for the Network will continue to be informed by the evolving needs of our target population. Phase 2 entails the formative process evaluation, the data collection period for which is from May 2022 to May 2023. Data analysis and the dissemination of a freely available web-based report will take place in summer 2023. For the final phase of the project, the summative impact assessment, data collection will take place between September 2023 and September 2024. Following this period, the results of the evaluation will be made publicly available through the Network, in addition to being published and presented at academic conferences.

## Discussion

The anticipated main findings of the proposed evaluation are that the Student Mental Health Network will fill existing gaps in the mental health promotion supports currently provided at postsecondary institutions across Canada. In doing so, we hypothesize that engagement with the Network will result in improvements in mental health education, the ability to develop strong social support Networks, and awareness of available help-seeking resources. Further, improving students’ access to effective upstream mental health promotion supports may contribute toward alleviating the long wait times and general overwhelm that downstream mental health services currently face by increasing students’ awareness of the spectrum of available services from low- to high-intensity options and helping students access services that align with their level of mental health need.

The accessibility of the Network is vital to its utility and is an important strength of the project; it is available to all postsecondary students on the internet at no cost. The “for-students, by-students,“ participatory approach to development ensures that content is meaningful and tailored to the target audience while maintaining relevance and timeliness. Given the nation-wide accessibility of the Network and recruitment for the participatory research design, we hope to obtain a wide sample variation spanning postsecondary institutions across the country. This may allow for the identification of inequities in the outcomes of interest between regions. Further, relationships between sociodemographic variables and these outcomes of interest will be investigated, and findings may be used to tailor the development of future website content to combat identified inequities. Lastly, although the Network takes an upstream health promotion-focused lens, it also serves to connect a generation of students with an interest in mental health and well-being. Through the provision of information and facilitation of social connection, the Network may empower collective action to advocate for institutional, political, and service-level change in current approaches to postsecondary mental health—“for-students, by-students.” The Network is a first-of-its-kind initiative that aims to fill the urgent, evidence-supported gaps in student mental health through an innovative, collaborative design.

Importantly, the overarching goal of the Network is to provide opportunities for knowledge translation and exchange that will support postsecondary students’ mental health and well-being. To that end, a technical report detailing evaluation results as well as a summary of Network resources will be made publicly available on an annual basis, with reports released in August. Network users will be invited to sign up for a mailing list, through which they will receive a copy of each report as it becomes available. We will also seek to share our annual reports through organizational connections such as the Best Practices Network [46] and the Center for Innovation in Campus Mental Health [47]. Transparency in the communication of the results of our evaluation of the Network will contribute toward a positive community impact by allowing institutions to examine the efficacy of the Network as a universal mental health promotion initiative. Results may also inform improvements to campus-specific mental health promotion and mental illness prevention resources and programming.

The novelty of the Network is both a strength and a limitation, as there is currently no set process for developing or evaluating this form of content. It is challenging to predict what uptake of the site will look like, but website usage statistics and social media interactions have demonstrated that the Network is receiving increasing attention from students since it was made public in May 2022. In terms of the analysis, it is not possible for residual confounding to be eliminated and observed changes in outcomes will not be attributable to the Network alone. There will also inevitably be some loss to follow-up, although a rolling recruitment strategy and raffle entry with each completion of the survey will be used to limit the effect on study power. Content on the Network is in English, and thus may be less accessible to French-dominant parts of Canada, international students, immigrants, and other groups who do not speak English as their first language. Further, not all students have equitable access to technology and social media or the time to navigate the Network and participate in voluntary surveys, which may result in some degree of selection bias.

### Conclusion

Strategies to address postsecondary mental health have emerged at the national, provincial, and institutional levels [10]. Over the past decade, many postsecondary institutions have attempted to improve students’ mental health by offering both upstream and downstream mental health supports, though typically the majority of resources are placed downstream [5,10]. Bolstering upstream supports like mental health promotion may help alleviate the current bottleneck observed at the downstream service level. Many students default to seeking help from high-intensity downstream services (ie, counseling and pharmacotherapy) when their level of mental health need does not necessarily align with this level of help seeking (ie, those who have not yet become symptomatic or reached a clinical threshold). There is a need to increase both the provision and awareness of lower-intensity upstream supports available on campuses to provide options for students experiencing a lower level of mental health need. This will in turn alleviate the pressure on downstream campus mental health services and ensure more timely downstream care for students who require it [29,30].

The Student Mental Health Network is a first-of-its kind web-based one-stop shop for mental health education and evidence-based resources, targeted specifically toward postsecondary students and uniquely developed using a participatory “for-students, by-students” approach. The Network will fill gaps in existing upstream mental health promotion supports provided by postsecondary institutions across the country, including eliminating fees and facilitating universally accessible mental health promotion for postsecondary students across Canada. The evaluation described here will allow us to assess the feasibility, acceptability, and utility of the Network, in addition to its effectiveness in improving the 3 primary outcomes of interest—mental health education, perceived social support, and help-seeking behavior—which are each proven predictors of positive mental health outcomes among students. This initiative has the potential to improve the mental health and well-being of postsecondary students across the country by providing access to the tools and resources required to build resiliency and respond to changes in one’s mental health.

## Appendix

Multimedia Appendix 1 Logic model.

Multimedia Appendix 2 Included measures.

## Abbreviations

LOI: letter of information
MHL: mental health literacy
OMS-WA: Opening Minds Scale – Working Attitudes
REB: Research Ethics Board
SES: socioeconomic status
TCPS2: Tri-Council Policy Statement 2

## Data Availability

Evaluation data will be made publicly available in a repository following the data collection period.
