# Supplementary material for: Canada’s Student Mental Health Network: Protocol for a Comprehensive Program Evaluation
Source: JMIR Res Protoc. 2023 Jun 22;12:e41521. doi: 10.2196/41521 (PMC10337409; doi:10.2196/41521)
Supplement: Multimedia Appendix 1 [file resprot_v12i1e41521_app1.docx]

***Supplementary File A:*** *Measurement Index*


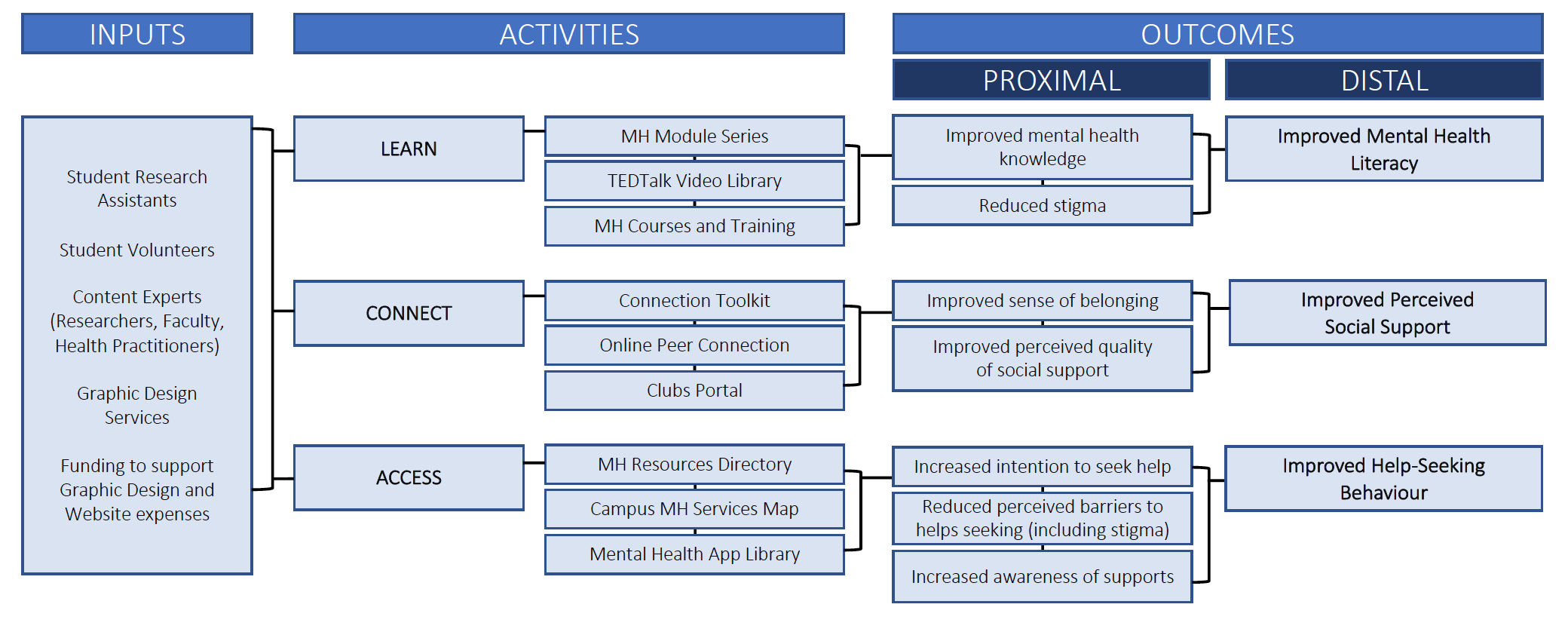
***Notes:*** *"Inputs” for this evaluation include resources (e.g., student Research Assistant to support logistics of the project) and funding to support operations (e.g., website domain name, graphic design, etc.)*
