# Supplementary material for: Canada’s Student Mental Health Network: Protocol for a Comprehensive Program Evaluation
Source: JMIR Res Protoc. 2023 Jun 22;12:e41521. doi: 10.2196/41521 (PMC10337409; doi:10.2196/41521)
Supplement: Multimedia Appendix 2 [file resprot_v12i1e41521_app2.docx]

**Website Engagement and User Experience**

***Website Attributes Questionnaire.*** A recent literature review identified a series of key attributes that affect user experience with a website (Allison et al., 2019). For the purposes of the evaluation of the Network, we will develop a custom Website Attributes Questionnaire as recommended, measuring four attributes most relevant to the network were selected and adapted: appearance, content, interactivity, inclusivity/representation, and accessibility. Participants will be asked to rank their level of agreement with a series of questions (e.g., “I would recommend the SMHN website to a friend or colleague.”, “The content is relevant to me as a student”, and “The content is trustworthy.” Using a 5-point Likert scale ranging from 1 (strongly disagree) to 5 (strongly agree). The web attributes questionnaire can be used for both formative and summative components of the proposed evaluation.

***Website Analytics****.* A recent literature review of electronic mental health interventions in randomized controlled trials revealed that more than half of studies did not state their methods for recording web usage, and only 5% checked the reliability of these data collection methods (Koneska et al., 2020). Google Analytics is a free service and has shown to be an effective method of process evaluation for electronically- delivered projects, such as the Network. In sum, Google Analytics provides data to answer three key questions in terms of process evaluation of an Internet-delivered intervention: (i) how do visitors behave?; (ii) where do visitors come from? and (iii) what content are visitors exposed to? These three allow Google Analytics to be used as an evaluation method for online interventions (Crutzen et al., 2013). Data collected through Google Analytics will be used to measure dissemination of the network, engagement, and participants’ navigational behaviour. Data on the total number of users, new users per week, average time spent on the website, geographical distribution of users by country, number of views per page, and the origin of users (social media, returning, or new user) will be included.

**Mental Health**

***Perceived Stress Scale (PSS-4).*** The PSS-4, derived from the original PSS, composed of 10 items, is designed to evaluate overall perceived stress level (Cohen et al., 1983). It is a self-report scale with scale items asking respondents how often they felt or thought a certain way within the past month, with response options ranging from 0 (never) to 4 (very often). A composite score, ranging from 0 to 16 is obtained by summing all responses after reverse coding the appropriate items. A higher score is indicative of higher levels of overall stress (Cohen et al., 1983). PSS-4 is based on psychometric principles and is considered to be sound (Cohen et al., 1983). The PSS-4 has demonstrated strong psychometric properties among samples of post-secondary aged youth, including…

***Kessler Psychological Distress Scale (K-6).*** The K6 is designed to measure levels of general psychological distress based on 6 questions asking participants if they had felt nervous, hopeless, restless, or fidgety; so depressed that nothing could cheer you up; that everything was an effort; and worthless in the past month. Response options range from 0 (none of the time) to 4 (all of the time), with a composite score ranging from 0 to 24 obtained by summing all responses (Ferro, 2019). Higher scores indicate higher levels of psychological distress. The scale K6 is a robust predictor of severe mental illness among youth (Kang et al., 2015), particularly for mood and anxiety disorders (Chan & Fung, 2014). In a study of Canadian adolescents by Ferro, 2019, the K6 was found to be valid and reliable in an epidemiological sample of Canadian youth.

***Brief Post-Secondary Student Stressors Index (Brief-PSSI).*** The Brief PSSI is a 14-item, abbreviated version of the Post-Secondary Student Stressors Index, designed to evaluate the severity and frequency of stressors experienced by post-secondary students. The original 46-item PSSI has been validated in Canadian post-secondary student population (Linden et al., 2022; Linden & Stuart, 2019), demonstrating strong psychometric properties. Content validation and response processes evidence was derived from active student involvement throughout the development and refinement of the tool, with test-test reliability to established instruments (Linden & Stuart, 2019). Despite its effectiveness in the postsecondary student population, the scale is long and in contexts such as ours, shorter scales are preferred. The Brief-PSSI is currently undergoing validation, with a pilot population of university students recently having completed the 14-item version. Due to the good psychometric properties of the PSSI, it is expected for the Brief-PSSI to exhibit similar properties.

**Mental Health Literacy**

***Mental Health Literacy Scale (MHLS - Adapted).*** Individual knowledge of mental health literacy, defined as an individual’s knowledge and beliefs about mental illness, mental health, the difference between the two as described in Keyes’ Mental Health Continuum, and knowledge of risk factors and causes of mental illness, treatment options, professional supports, and how to seek mental health information will be assessed using a brief, 10-item self-report created for this program evaluation. Attributes of mental health literacy were adapted from the Mental Health Literacy Scale (O’Connor & Casey, 2015) and supplemented to meet the needs of this evaluation. Participants will be presented with questions (e.g., “I am confident that I know where to seek information about mental illness?”) and asked to rate their level of agreement on a 5-point Likert scale ranging from 1 (strongly disagree) to 5 (strongly agree).

***Social Distance Subscale of OMS-WA Scale (Adapted).*** The OMS-WA is a 22-item scale originally designed for workplace environments with the purpose of assessing attitudes, stereotypes, and behavioural intentions towards individuals with mental illness (Dobson et al., 2019). These are measured on a 5-point Likert scale. A further study building upon this (Szeto, et al., 2021) adapted the scale to match the post-secondary environment with not many major changes being made (e.g., changing language that includes students/instructor rather than employees/employers, removing questions on dating in the workplace). This 23-item adapted multidimensional scale encompasses 5 subscales, one for each of the 5 dimensions of stigma. Of particular interest for our study is the subscale *desire* *for* *social* *distance*, which has been independently validated with an high Cronbach’s alpha (0.88-0.92), showing strong internal validity (Szeto, et al., 2021). An example of a statement from this subscale is *“I would be upset if someone with a mental illness always sat next to me in class.”*

**Social Support and Resilience**

***Multidimensional Scale of Perceived Social Support (MSPSS).*** Perceived social support will be evaluated using the MSPSS, a 12-item self-report measure developed to assess perceptions of received support from an individual’s family, friends, and significant other. Participants are presented with items relating to each source of social support (e.g., “My family really tries to help me”) and asked to rate their agreement with each statement on a 7-point Likert scale ranging from 1 (*very strongly disagree*) to 7 (*very strongly agree*) (Zimet et al., 1998). Higher scores indicate greater perceived social support from a specific source. The MSPSS has displayed good internal consistency across each subscale (αs = .81 to .98) as well as factorial validity, confirming the proposed three-subscale structure of the MSPSS (Zimet et al., 1990). Psychometric studies using this scale in students/adolescents confirm the reliability, validity, and utility of the MSPSS in this population (Canty-Mitchell & Zimet, 2000; Kong et al., 2015)**.**

***Connor-Davidson Resilience Scale (CD-RISC-10).*** The CD-RISC-10 is a 10-item, unidimensional self-report measure designed to assess an individual’s ability to cope with adversity and is a shortened version of the original 25-item scale. Items (e.g., “*I am able to adapt to change*”) are rated on a 5-point adjectival scale ranging from 0 (*not true at all*) to 4 (*true nearly all of the time*) (Campbell-Sills & Stein, 2007; Connor & Davidson, 2003). Responses are summed for a composite score, from 0-40, with higher scores indicating a higher level of resilience. The CD-RISC-10 has demonstrated strong internal consistency reliability and evidence for validity (including content evidence, relations to other variables, and internal structure) in a variety of studies (Campbell-Sills & Stein, 2007; Heritage et al., 2021) and has been implemented among a variety of post-secondary populations (e.g., Cheng et al., 2020; Singh & Yu, 2010; Smith et al., 2019).

***Canadian Campus Wellbeing Survey:*** the CCWS is a survey designed to help post-secondary institutions better support the wellbeing and mental health of their students composed of 9 core sections (student experience, mental health deficits, health service utilization/ help-seeking, physical health/health behaviors, academic achievement, substance use, nutrition, and sexual health. The framework was developed using a three-round Delphi survey where participants identified wellbeing and health behavior measurement priorities and indicators for the tool (CCWS Expert Panel Group et al., 2019). Responses for each sub question of the tool range from (“*strongly disagree*”) to (“*strongly agree*”). The CCWS was specifically designed for Canadian post-secondary students, which is the population that will be participating in our evaluation.

**Help Seeking**

***Barriers to Access to Care Evaluation (BACE, Clement et al. 2012).*** The BACE is a scale developed from items in existing scales, systematic item reduction, and feedback from a group of experts. It is a 30-item, 4-point self-report scale with responses ranging from 0 (*not at* all) to 3 (*a* lot). Items had acceptable test-test reliability, internal validity, internal consistency and acceptability (Clement et al., 2012; Silva et al., 2013). The scale includes a stigma subscale for assessing the stigma associated with receiving mental health care. This subscale was found to have good test-retest reliability, internal consistency, and content and construct validity. Clement et al (2019) concluded that the scale can be used to ascertain key barriers to access to mental health care which may help to identify potential interventions to increase care seeking and service use.

**The General Help Seeking Questionnaire (GHSQ; Deane et al., 2001).** The GHSQ is a measure of formal help seeking intentions for non-suicidal and suicidal problems using a matrix design. Respondents are asked two questions, however in our context we will only use the first: (“If you were having a personal or emotional problem, how likely is it that you would seek help from the following people?”) and then asked to rank their help-seeking intentions on a 7-point scale ranging from 1 (*extremely unlikely*) to 7 (*extremely likely*) for ten help source options, with higher scored indicating higher intentions. The GHSQ has been widely used across pre- and post-secondary student populations and was found to have satisfactory reliability and validity (Wilson et al., 2005).
